# Perioperative Challenges: Analysis of Surgical Complications in Screening Lung Carcinoma Patient

**DOI:** 10.7759/cureus.64700

**Published:** 2024-07-16

**Authors:** Miljana Poparić, Jovan Baljak, Ivan Ergelašev

**Affiliations:** 1 Oncology, Faculty of Medicine, University of Novi Sad, Novi Sad, SRB; 2 Department of Surgery, Faculty of Medicine Novi Sad, University of Novi Sad, Novi Sad, SRB

**Keywords:** air leak, length of hospital stay, screening, surgical complications, video-assisted thoracoscopic surgery (vats)

## Abstract

Introduction

In September 2020, the Institute for Pulmonary Diseases of Vojvodina (IPBV) started a lung cancer screening program using low-dose computed tomography (LDCT). Video-assisted thoracic surgery (VATS) lobectomy is the most effective treatment for early-stage lung cancer. However, the frequency of postoperative complications in VATS anatomical lung resections among patients enrolled in the screening program has not been adequately studied. This study aims to compare the frequency of surgical complications and length of hospital stay between patients enrolled in the screening program and a control group.

Methods

Retrospective, observational, monocentric, non-randomized study was conducted at the IPBV in Sremska Kamenica. The study included patients with a confirmed diagnosis of lung cancer who underwent anatomic pulmonary resection with mediastinal lymphadenectomy for therapeutic purposes. The patients were divided into two groups: the first group consisted of 34 patients who participated in the lung carcinoma screening program, while the second control group consisted of 102 patients. Over the past three years, all patients identified with nodules suspicious of malignancy during the screening program were sequentially enrolled in the screening group. For the control group, patients were selected based on a matching process to ensure valid statistical comparisons with the screening group. They were matched in a 3:1 ratio with patients from the screening group based on criteria including gender, disease stage, pathohistological type of cancer, tumor, node, and metastasis (TNM) stage of the disease, and degree of surgical resection. Patients were monitored for demographic parameters, smoking status, presence of comorbidities and prior oncological diseases, pulmonary function parameters, level of pre-operational risk, the number of lymph nodes removed by biopsies, spread through alveolar spaces (STAS), and the occurrence of complications after surgery (infection, bleeding, air leak, presence of adhesions), re-drainage, and length of hospital stay.

Results

The patients in the screening group had a higher incidence of infections, bleeding, prolonged air leak, and required re-drainage after surgery compared to the control group. Patients from the screening program with a high operative risk, prolonged air leak, and pleural adhesions had a statistically significant higher hospital stay longer than the control group.

Conclusions

This research emphasizes the importance of screening programs for detecting lung cancer in the early stages. However, it also highlights the need for further research to reduce surgical complications and improve therapeutic interventions for patients in the screening program.

## Introduction

Lung cancer is one of the most common cancers and is the leading cause of death among cancers, both in the world and in Serbia [1]. According to the Global Cancer Observatory (GLOBOCAN) data for 2022, there were approximately 6,879 (16.4%) newly diagnosed cases of lung cancer in Serbia [2]. The mortality of patients with lung cancer accounts for 25% of the total number of deaths related to malignant diseases [1]. The main risk factor for the development of cancer is active and passive smoking [3]. In addition to smoking, genetic and environmental factors, previous occurrences of lung disease, and occupational exposure are responsible for the development of lung cancer [4].

Better survival for lung cancer patients is possible by establishing a diagnosis at an early stage of the disease. The five-year survival of lung cancer patients in stage I ranges from 71% to 90%, while in stage IV, it is 5% to 10%. In our country, approximately 60% of lung cancer patients are diagnosed at stage IV [5]. Because of the enormous public health impact that lung cancer has on our country, in September 2020, a lung cancer screening program using low-dose computed tomography (LDCT) in the high-risk population in Vojvodina started. The program "Early Detection of Lung Cancer" is organized and financed by the Provincial Secretariat for Health Care, Vojvodina, Republic of Serbia. Lung cancer screening with LDCT in a high-risk population (active, former smokers) has been demonstrated to be an effective method of secondary prevention [1].

The therapeutic modality in the management of lung cancer treatment includes a multimodal way of treatment: surgery, radiotherapy, chemotherapy, biological therapy, immunotherapy, and palliative patient care. Therapy has an individual approach based on a specific health condition, and the main aim of surgical treatment is complete healing [6]. Anatomic lung resection represents the gold standard in treating lung cancer in the early stages [7]. For patients with stages I, II, and IIIA, the use of surgical treatment is recommended [8]. Surgical resection, including thoracotomy and minimally invasive surgery (MIS), is the preferred treatment for lung cancer. Since the video-assisted thoracic surgery (VATS) lobectomy was first performed in the early 1990s, VATS has become widely accepted in thoracic surgery. In recent years, VATS has been recognized for its advantages over MIS in the treatment of lung cancer, such as less bleeding, improved recovery, a shorter length of hospital stay, and lower mortality. Clinical trials comparing VATS with conventional thoracotomy have shown that VATS has superior perioperative outcomes and improved long-term survival [9].

Postoperative complication is any complication that occurs within 30 days after surgery [7]. The frequency of postoperative complications associated with lung resection for cancer is 10%-40%. The frequency of postoperative complications depends on age, smoking status, the presence of comorbidities, and the type of surgical intervention [8]. Prolonged air leaks, the appearance of pneumonia, bronchopleural fistula, atelectasis, and pneumothorax are frequent respiratory complications. Atrial fibrillation, ventricular tachycardia, and myocardial infarction represent cardiovascular complications. Non-recognition and inadequate treatment can lead to severe complications, which, in the last case, can be fatal [10].

This research aims to compare the frequency of occurrence of comorbidities, the length of hospital stay, and the frequency of occurrence of surgical complications in patients from the program for early detection of lung cancer compared to the control group who underwent anatomical lung resection with mediastinal lymphadenectomy for therapeutic purposes. Also, the research aims to determine the relationship between the occurrence of surgical complications after surgery and the length of hospital stay.

## Materials and methods

This study was performed at the Institute for Pulmonary Diseases of Vojvodina (IPBV) in Sremska Kamenica. The obtained data were collected from the medical documentation of the information system of the IPBV. The collection of data and the writing of a scientific research paper were approved by the Ethics Committee and the Professional Council of the IPBV. A retrospective, observational, monocentric, non-randomized study includes 136 patients of both genders who were hospitalized for IPBV from September 2020 to October 2023.

The study included patients with a confirmed diagnosis of lung cancer who were divided into two groups. The first group consisted of 34 patients who participated in the screening program, while the second group consisted of patients from the control group. Over the past three years, all patients identified with nodules suspicious of malignancy during the screening program were sequentially enrolled in the screening group. These patients underwent LDCT, identifying suspicious, enlarged thoracic nodules. Subsequently, a bronchoscopy was performed for diagnostic purposes, confirming the diagnosis of bronchial cancer. Following the early-stage diagnosis, these patients underwent surgical anatomical resection with lymphadenectomy for therapeutic purposes. The control group consisted of patients with a confirmed diagnosis of bronchial cancer who also underwent surgical anatomical resection with mediastinal lymphadenectomy. For the control group, patients were selected based on a matching process to ensure valid statistical comparisons with the screening group. They were matched in a 3:1 ratio with patients from the screening group based on criteria including gender, disease stage, pathohistological type of cancer, tumor, node, and metastasis (TNM) stage of the disease, and degree of surgical resection. Screening of lung cancer using LDCT was carried out in IPBV. All patients in the study were aged 50-74 years, former smokers (stopped smoking 10 years ago), and active smokers with 20-30 years of smoking with additional risk factors. For all patients, anatomic lung resection with lymphadenectomy was performed for therapeutic purposes. Patients were monitored for demographic parameters, smoking status, presence of comorbidities and prior oncological diseases, pulmonary function parameters (vital capacity, forced vital capacity, forced expiratory volume in the first second, peak expiratory flow, intrathoracic gas volume), level of pre-operational risk, the number of lymph nodes removed by biopsies, spread through alveolar spaces (STAS), and the occurrence of complications after surgery (infection, bleeding, air leak, presence of adhesions), re-drainage, and length of hospital stay.

Data collection and statistical processing were performed using SPSS Statistics (IBM SPSS Statistics for Windows, IBM Corp., Released 2019, Version 26.0, Armonk, NY). The χ^2^ test was used to compare the frequency of surgical complications about theoretical probabilities between patients from screening and the control group. The Mann-Whitney U test was used to compare the length of hospital stay between the screening and control groups. All differences were considered statistically significant at the 0.05 or 0.001 significance level (p < 0.05; p < 0.001).

## Results

A total of 136 patients were included in the study: 68 (50%) males and 68 (50%) females. The average age of patients in the screening program is 67 ± 6.9 years, while the patients in the control group are 66 ± 6.8 years. Patients from the screening program were all smokers (100%) with an average smoking age of 45 ± 8.8 years, while 82.35% of patients from the control group were smokers with an average smoking age of 32 ± 11.1 years. Of the total sample of patients with lung cancer, 67.24% of patients had adenocarcinoma, 23.52% squamous cell carcinoma (SCC), 5.88% large cell neuroendocrine carcinoma (LCNEC), and 2.94% small-cell carcinoma (SCC). Based on the TNM classification, the largest number of patients (47.05%) were classified as IA2 stage (T1bN0M0), while the smallest number of patients (5.88%) were classified as IA stage (TtisN0M0). Table 1 shows the average values of the patient's lung function parameters. No difference was observed between patients from the screening program and the control group in these parameters.

**Table 1:** Average values of lung function parameters in patients from the screening program and the control group FEV1: forced expiratory volume in the first second; FVC: forced vital capacity; ITGV: intrathoracic gas volume; PEF: peak expiratory flow; VC: vital capacity

Group	VC %	FVC %	FEV1 %	PEF %	ITGV %
Control group	110.58	112.92	93.07	90.78	145.61
Screening group	112.04	114.1	93.31	92.74	143.09

**Figure 1:**
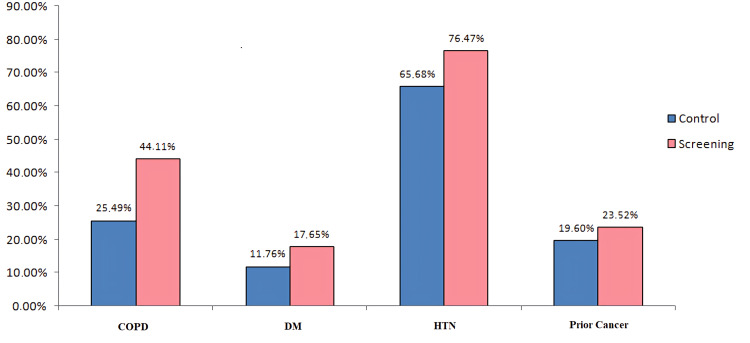
Frequency of comorbidities in lung cancer patients in the screening program compared to those in the control group receiving VATS lobectomy surgery COPD: chronic obstructive pulmonary disease; DM: diabetes mellitus; HTN: hypertension; VATS: video-assisted thoracic surgery

Figure 1 shows the percentages of comorbidities in patients with lung cancer in the screening program compared to those in the control group. Patients included in the screening program have a statistically significantly higher incidence of COPD than the control group of patients (χ^2^_(1.136)_ = 4.202; p < 0.05). No statistically significant difference in frequency was observed for the occurrence of other comorbidities.

**Figure 2:**
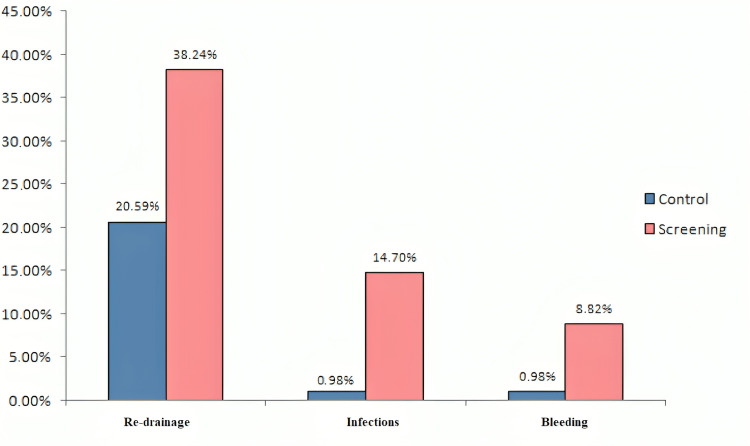
Frequency of surgical complications in patients with lung cancer in the screening program compared to those in the control group receiving VATS lobectomy surgery VATS: video-assisted thoracic surgery

Figure 2 shows the percentages of postoperative surgical complications after anatomical resection with mediastinal lymphadenectomy, which was performed for therapeutic purposes in patients who were diagnosed with lung cancer in the screening program compared to those in the control group. Patients included in the screening program have a statistically significantly higher frequency of surgical complications: re-drainage (χ^2^_(1.136)_ = 4.235; p < 0.05), infections (χ^2^_(1.136)_ = 11.391; p < 0.01), and bleeding (χ^2^_(1.136)_ = 5.495; p < 0.05) after surgery compared to patients of the control group.

**Figure 3:**
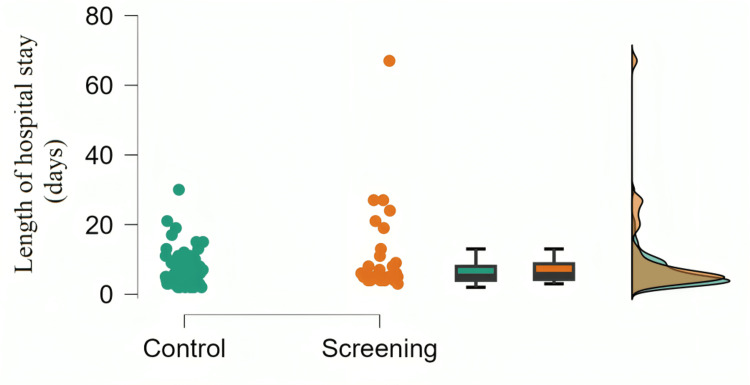
Length of hospital stay of patients from the screening program and the control group after anatomic lung resection with mediastinal lymphadenectomy

Patients with lung cancer from the screening program (10.23 ± 12.1 hospital days) have a statistically significantly higher number of hospital days compared to the control group (6.35 ± 4.47 hospital days) who receiving VATS lobectomy surgery (W =13,226.5; p < 0.05) (Figure 3).

Table 2 shows the percentages of patients depending on operational risk and air leaks in patients with lung cancer from the screening program and the control group who received anatomical resection surgery with mediastinal lymphadenectomy.

**Table 2:** Operational risk and air leaks of patients from the screening program and the control group

	Control group						Screening group					
Operational risk	Low		Medium		High		Low		Medium		High	
	33	32.35%	47	46.07%	22	21.56%	8	23.52%	20	58.82%	6	17.64%
Air leak	No		Small		Prolonged		No		Small		Prolonged	
	62	60.78%	31	30.39%	9	8.82%	22	64.70%	6	17.64%	6	17.64%

**Figure 4:**
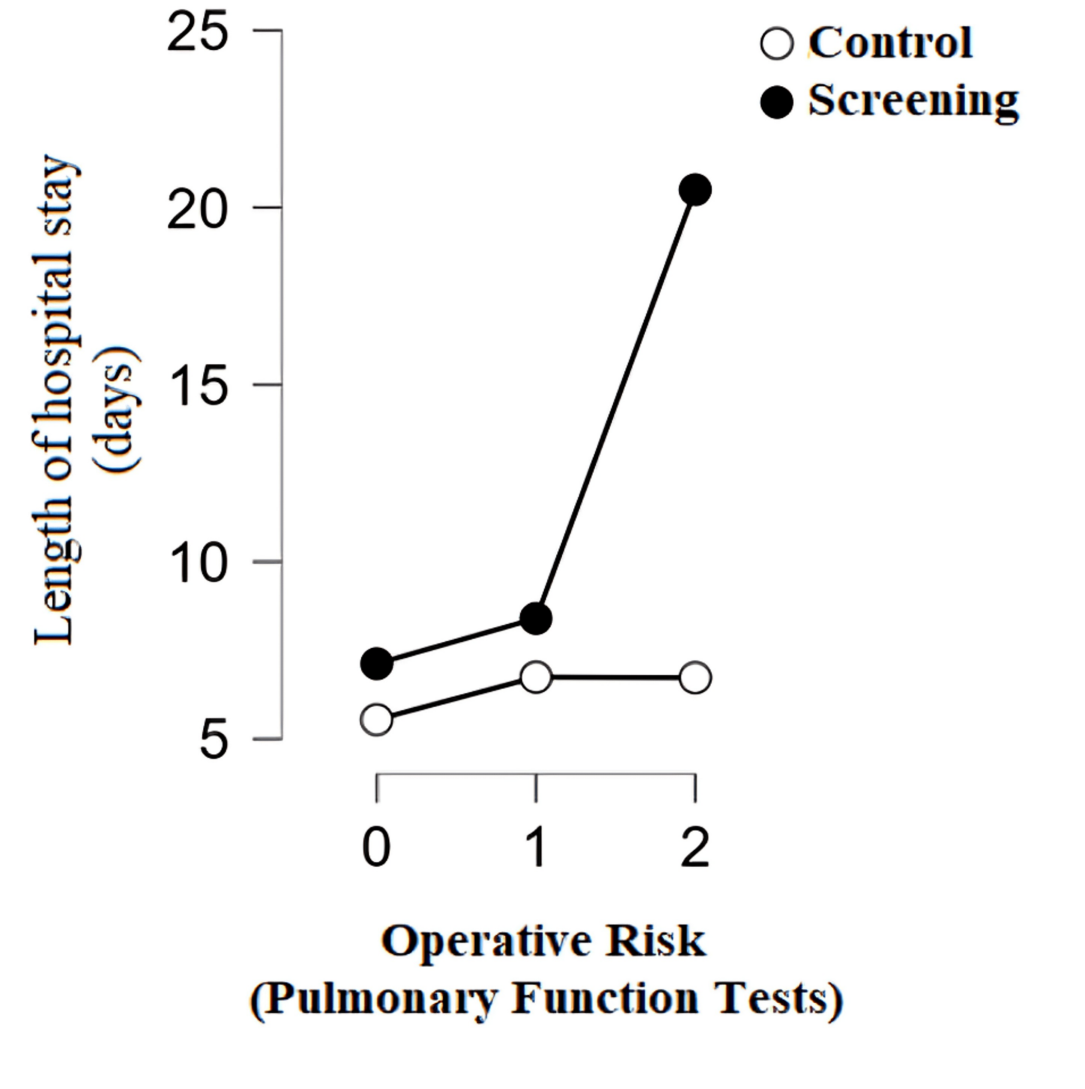
Length of hospital stay of patients depending on the operational risk by group 0: no risk; 1: medium risk; 2: high risk

Patients from the screening program with a high risk for surgery, which is predicted based on lung function parameters, had a statistically significantly higher number of hospital days than the control group with a high risk for surgery after anatomic lung resection (p < 0.001) (Figure 4). 

**Figure 5:**
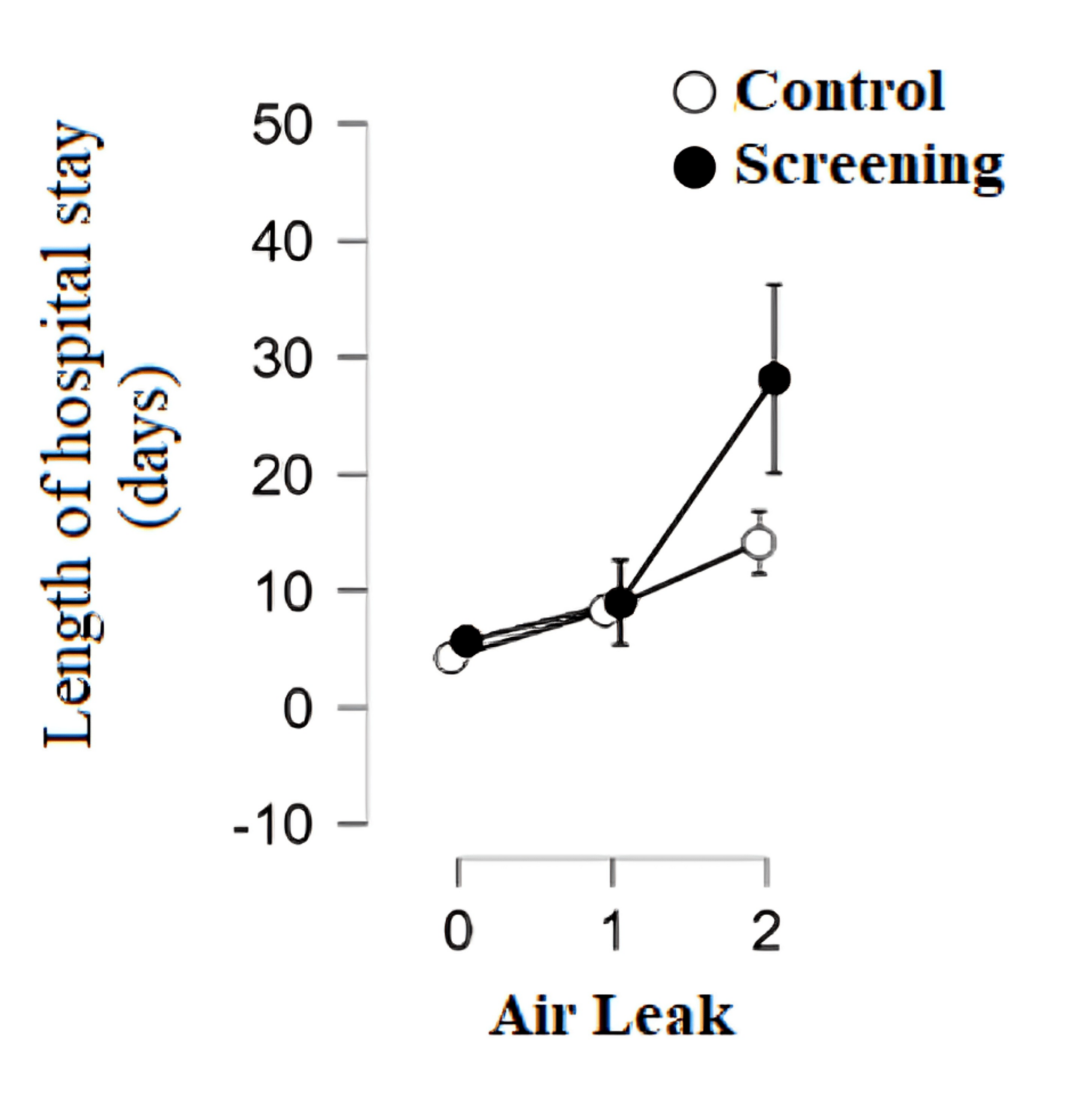
Length of hospital stay depending on air leaks by group 0: no loss; 1: moderate loss; 2: prolonged loss

This study showed a statistically significant difference in the length of hospital stays depending on air leaks. Prolonged air leaks were considered any air leaks that persisted for more than seven days after the operation. Patients with prolonged air leaks as a respiratory complication after surgery have a statistically significantly higher number of hospital days compared to patients without air leaks in both groups (p < 0.001). Patients from the screening program with prolonged air leaks (2) had a statistically significantly higher number of hospital days than the control group with prolonged air leaks (p < 0.001) (Figure 5). Results showed a statistically significant positive correlation between the length of hospital stay and air leaks in the group of patients from the screening program (rho = 0.538; p < 0.001) and the control group (rho = 0.630; p < 0.001).

**Figure 6:**
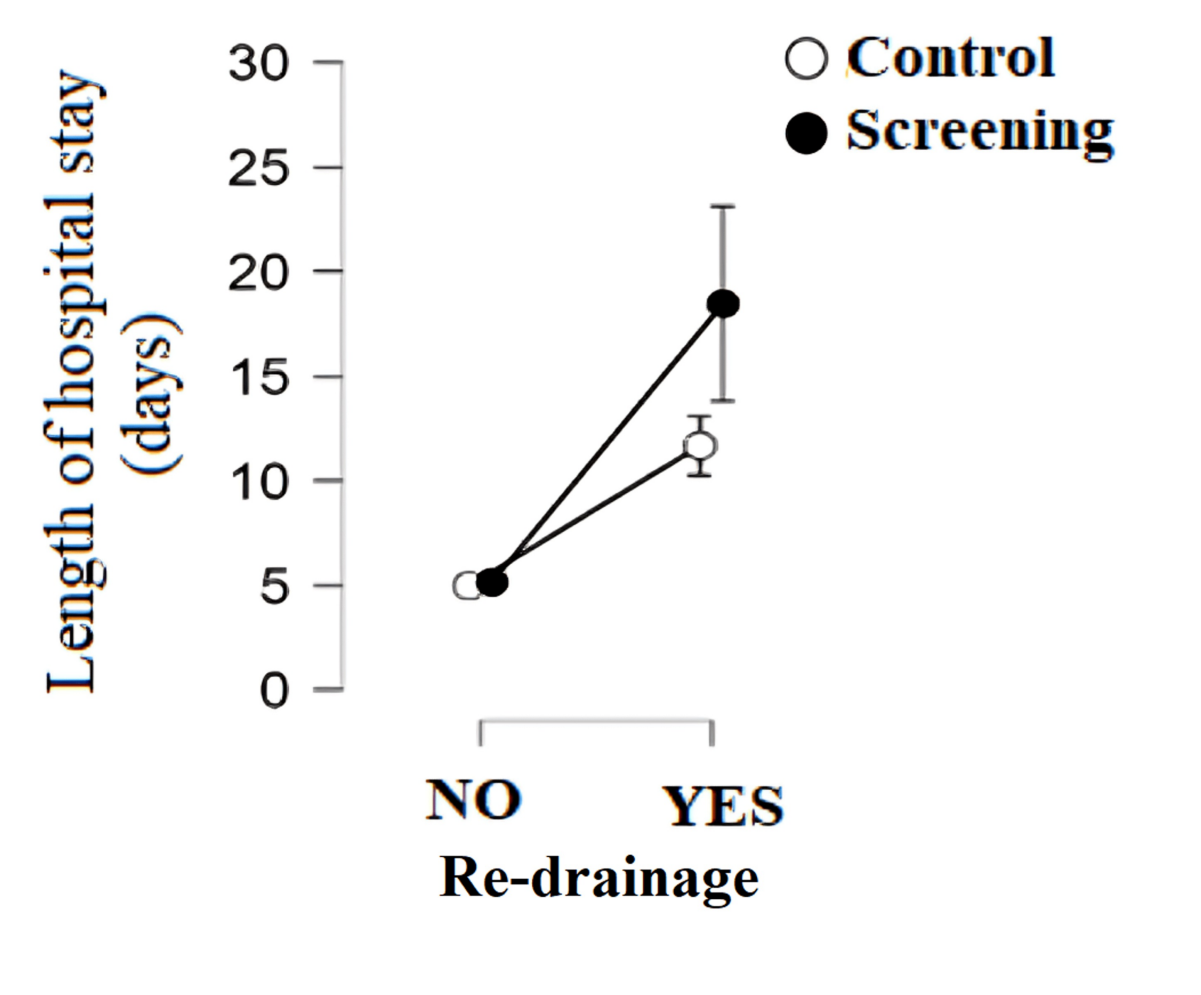
Length of hospital stay depending on the performance of re-drainage after anatomic lung resection by group

There is a statistically significant difference in the length of hospital stay depending on whether re-drainage was performed because of the occurrence of respiratory complications after surgery. Patients who underwent re-drainage had a statistically significantly higher number of hospital days compared to patients who did not undergo re-drainage in both groups (p < 0.001). Patients from the screening program who underwent re-drainage had a statistically significantly higher number of hospital days than the control group (p < 0.001) (Figure 6). Patients with prolonged air leaks have a higher frequency of occurrence of re-drainage after surgery compared to patients with other stages of air leaks (χ^2^_(2.136)_ = 58.395; p < 0.001).

**Figure 7:**
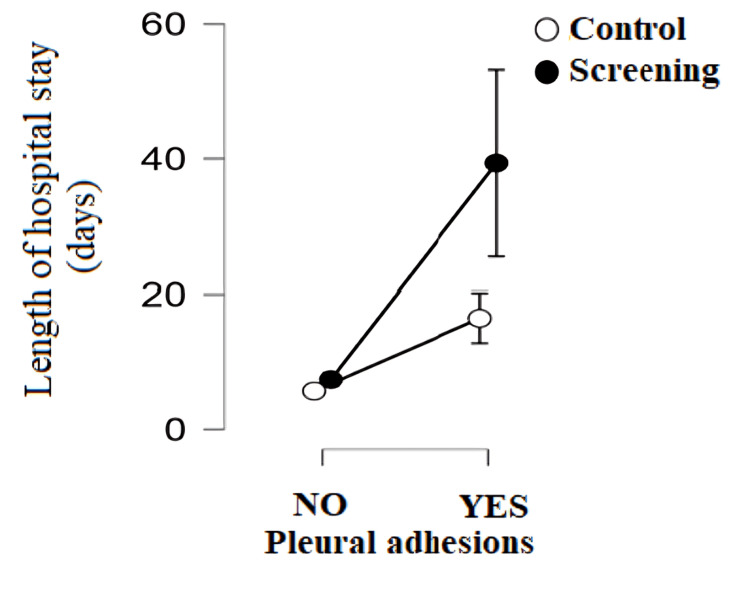
Length of hospital stay depending on the presence of adhesions before surgery by group

The study showed a statistically significant difference in the length of hospital stay depending on the presence of pleural adhesions. The formation of adhesions or connective tissue in the organs inside the chest is a consequence of the inflammatory process before surgery. Patients in whom the appearance of pleural adhesions occurred before surgery had a statistically significantly higher number of hospital days compared to patients in whom there were no recorded adhesions in both groups (p < 0.001). Patients from the screening program in whom the presence of adhesions was recorded had a statistically significantly higher number of hospital days than the control group in which the presence of adhesions was also recorded (p < 0.001) (Figure 7). There is no statistically significant difference in the incidence of tumor STAS between patients from the screening program and the control group (χ^2^_(1.136)_ = 2.603; p > 0.005).

## Discussion

The start of the implementation of the program for early detection of lung cancer by LDCT dates from September 2020 at the IPBV. Advances in medical research and technological innovations have contributed to more accurate diagnostic and therapeutic strategies, especially in the early stages of cancer. This program made it possible to detect lung cancer at an early stage in 34 patients, for whom it served as the basis for curative surgical intervention.

The analysis of the results of samples from patients undergoing anatomical resection with mediastinal lymphadenectomy provides valuable insight into the variety of histological types of cancer and their TNM classification. The frequency of adenocarcinoma (67.24%) is statistically significantly higher compared to the expected frequency in the population, while small cell lung carcinoma (2.94%) was statistically significantly lower compared to the expected frequency in the population (χ^2^ = 49.98; p < 0.01). The majority of patients are classified in the early stages (IA2), but the smaller number of patients identified in the later stages of the disease should not be ignored. This emphasizes the importance of screening procedures and early diagnosis to improve treatment outcomes [1].

Patients included in the screening program have a statistically significantly higher incidence of COPD than the control group of patients. These data can explain why patients from the screening program have a higher frequency of postoperative complications because of a higher frequency of COPD compared to the control group. Okada et al. showed a relationship between the development of pulmonary postoperative complications and COPD but with a non-significant trend [11]. Studies published so far indicate that patients with COPD have a higher frequency of pulmonary postoperative complications and overall complications [12-15]. Contrary to these results, the study by Benker et al. indicated that none of the analyzed comorbidities, even COPD, was an independent predictor of postsurgical complications after anatomical lung resection [10].

Postsurgical complications after VATS anatomical lung resections in patients from the screening program have not been sufficiently published. This study found that patients who participated in a screening program had a higher hospital stay compared to a control group who underwent VATS lobectomy. The reason for this result is that patients in the screening program have a higher incidence of infections and bleeding after surgery, as well as a higher need for re-drainage due to incomplete lung re-expansion compared to patients in the control group, which prolongs their hospital stay. Many studies published so far have confirmed that pulmonary complications are the most common postsurgical complications that prolong hospital stays [10, 11]. This result confirms the importance of screening for early detection of lung cancer but, at the same time, indicates the need for further research to reduce surgical complications and improve therapeutic interventions in these patients.

Patients from the screening program with high operational risk, prolonged air leaks, and the presence of adhesions before and after surgery had a statistically significantly higher hospital stay than the control group. The study by Bedat et al. confirmed that high operational risk is significantly associated with the occurrence of postoperative complications and, indirectly, a higher hospital stay [8]. Patients from the screening program had a significantly higher incidence of prolonged air leaks (17.60%) compared to the control group (6.60%). Because there is a strong positive relationship between air leaks and length of hospital stay, this explains why patients from the screening program had a higher hospital stay compared to the control group. Today, an estimated 8%-15% of patients experience prolonged air leaks after lobectomy, which requires careful preoperative patient education about possible consequences, including prolonged hospital stay or use of a drain connected to a Heimlich valve after discharge [16]. This complication, although often the only one after surgery, has potentially serious consequences such as prolonged hospital stays, increased financial expenses, and a higher risk of other medical problems [17]. The main complications that often occur in connection with prolonged air leaks are infections in the pleura and lungs, which in 90% of cases represent serious complications [18].

Adhesions in the pleural space represent scarring changes that can fix the visceral pleura to the parietal pleura. In addition to affecting the pleural space, adhesions can also damage the subpleural lung tissue, reducing its elasticity and increasing the risk of prolonged air leaks after its excision. Previous research suggests an association between the presence of adhesions and prolonged air leaks, which further prolongs hospital stays [19]. Patients who have prolonged air leaks require re-drainage more often than expected. This may explain the prolonged hospital stay of patients undergoing re-drainage, as there is a direct relationship between prolonged air leaks and re-drainage.

The research highlights the link between prolonged air leaks, postoperative complications, and prolonged hospital stays, highlighting the need for further research to improve therapeutic approaches and reduce surgical complications. Although surgical complications impair the patient's satisfaction with the treatment, the final functional and oncological results are crucial and shape the overall impression that patients form.

## Conclusions

This research determined that patients from the screening program have a statistically significantly higher incidence of COPD than the control group. Patients who participated in the screening program had a longer hospital stay compared to the control group, who underwent anatomic resection with mediastinal lymphadenectomy. This study showed a higher incidence of post-operative re-drainage, infection, and bleeding as surgical complications in patients in the screening program, which prolonged their hospital stay compared to patients in the control group. Higher incidences of prolonged air leaks, adhesions, and postoperative re-drainage in patients from the screening program accounted for prolonged hospital stays in those patients compared to the control group. This study underscores the profound impact of effective screening programs in providing equitable access to healthcare interventions, particularly for patients from lower social backgrounds, potentially saving lives through timely medical evaluation and treatment, where each case represents a potentially saved life.
