# Correction: Perioperative Challenges: Analysis of Surgical Complications in Screening Lung Carcinoma Patient

**DOI:** 10.7759/cureus.c229

**Published:** 2025-07-09

**Authors:** Miljana Poparić, Jovan Baljak, Ivan Ergelašev

**Affiliations:** 1 Oncology, Faculty of Medicine, University of Novi Sad, Novi Sad, SRB; 2 Department of Surgery, Faculty of Medicine Novi Sad, University of Novi Sad, Novi Sad, SRB

The institutional affiliation for author Ivan Ergelašev has been corrected as follows.

Previous affiliation: Clinic for Thoracic Surgery, Institute for Pulmonary Diseases of Vojvodina, Sremska Kamenica, SRB

Correct affiliation: Department of Surgery, Faculty of Medicine Novi Sad, University of Novi Sad, Novi Sad, SRB

